# A global lipid map of severe fever with thrombocytopenia syndrome virus infection reveals glycerophospholipids as novel prognosis biomarkers

**DOI:** 10.1128/mbio.02628-24

**Published:** 2024-11-13

**Authors:** Panpan Tian, Liwei Zhao, Guiting Zhang, Shixing Chen, Wanying Zhang, Mingrong Ou, Yidan Sun, Yuxin Chen

**Affiliations:** 1 Department of Laboratory Medicine, Drum Tower Hospital Clinical College of Nanjing University of Chinese Medicine, Nanjing, Jiangsu, China; 2 Nanjing Drum Tower Hospital, Affiliated Hospital of Medical School, Nanjing University, Nanjing, Jiangsu, China; 3 Department of Laboratory Medicine, Nanjing Drum Tower Hospital Clinical College of Nanjing Medical University, Nanjing, Jiangsu, China; 4 Department of Laboratory Medicine, Nanjing Pukou People’s Hospital, Nanjing, Jiangsu, China; University of Colorado School of Medicine, Aurora, Colorado, USA; Colorado State University, Fort Collins, Colorado, USA

**Keywords:** SFTS, global lipidomics, glycerophospholipid, novel prognosis biomarkers

## Abstract

Severe fever with thrombocytopenia syndrome (SFTS) is a rapidly progressing infectious disease caused by a novel bunyavirus characterized by high fever, thrombocytopenia, and multiple organ damage. While lipids play an important role in viral infections, the specific alterations in lipid metabolism during SFTSV infection remain unclear. This study aimed to elucidate the global lipid metabolic profiles of SFTS patients with mild, severe, and fatal outcomes. A total of 60 SFTS patients, consisting of 30 mild, 15 severe and 15 fatal patients, and 30 healthy controls, were enrolled for the investigation of global lipidomics in serum using liquid chromatography-tandem mass spectrometry (LC-MS/MS). Our findings revealed global alterations in the lipid signature induced by SFTSV infection and further confirmed that the glycerophospholipid metabolism pathway was profoundly affected during the progression of mild, severe, and fatal outcomes in SFTS patients. Importantly, LysoPC (20:0) and LysoPC (P-16:0) are strongly correlated with the clinical parameters of SFTSV infection. Furthermore, we demonstrated the substantial prognostic value of LysoPC (20:0) and LysoPC (P-16:0) by receiver operating characteristic (ROC) curve analysis, providing evidence for their remarkable value as prognostic biomarkers for predicting SFTS clinical outcomes. In particular, LysoPC (20:0) and LysoPC (P-16:0), along with APTT, yielded superior prognostic performance for fatal SFTS [area under the curve (AUC) = 98.4%], outperforming routine clinical parameters. Collectively, our findings revealed the lipidomic landscape after SFTSV infection, which offers new insights into the mechanisms of SFTS disease progression and suggests that targeting lipid metabolism may serve as a potential therapeutic strategy.

**IMPORTANCE:**

This study systematically investigated the lipid landscape profile of SFTS-infected patients with different clinical outcomes. Our results revealed a global alteration in the lipid signature, particularly the glycerophospholipid metabolic pathway, induced by SFTSV infection. Notably, LysoPC (20:0) and LysoPC (P-16:0) presented remarkable prognostic value as novel biomarkers for SFTSV infection and may contribute to the prognosis of SFTS progression and appropriate interventions.

## INTRODUCTION

Severe fever with thrombocytopenia syndrome (SFTS) is an emerging tick-borne viral disease caused by the SFTS virus (SFTSV), which belongs to the *Bunyaviridae* family. Initially identified in China in 2009 (1), SFTSV infection is characterized by acute fever, thrombocytopenia, leukopenia, and in severe cases, hemorrhagic fever and multiple organ failure, with a high fatality rate ranging from 12% to 50% (2–4). Human-to-human transmission has been reported in familial and hospital settings, mainly through direct blood exposure (5). SFTS has been designated as a priority pathogen by the World Health Organization (WHO) due to its potential for causing outbreaks and its high mortality rate (6). Despite the continuously expanding geographic distribution of SFTS cases, no approved vaccines or specific antiviral treatments are currently available (7–9). Therefore, a better understanding of the physiopathology associated with disease progression is needed to identify biomarkers and effective antiviral therapies to alleviate disease symptoms and to mitigate long-term morbidity.

Lipids play crucial roles in viral infection, influencing various stages of the viral lifecycle and host immune responses (10). SFTSV, like many viruses, enters host cells by exploiting lipid rafts enriched in cholesterol and sphingolipids. These lipid rafts act as gateways for SFTSV entry, facilitating the fusion of the virus with host cell membranes through the clustering of viral entry receptors such as CCR2 (11, 12). Lipids are essential components of viral membranes and replication organelles. SFTSV replicates its RNA genome in specialized compartments within host cells (13, 14), which may involve alterations in lipid metabolism for viral replication and assembly. Our previous study showed that SFTSV-infected patients exhibited alterations in serum lipid metabolism parameters with lower levels of HDL cholesterol, LDL cholesterol, apolipoprotein A-I, and apolipoprotein B but elevated levels of triglycerides, suggesting that SFTSV may influence the serum levels of lipids, which could serve as potential biomarkers for predicting the virus infection process (15).

Among the myriad metabolites circulating in blood plasma, the lipidome represents a tightly regulated and precisely defined entity that is garnering increasing interest in investigating disease pathophysiology. The level of circulating lipids can serve as an indicator of metabolic changes associated with various conditions, including cancer, metabolic disorders, and neurodegenerative diseases (16–20). Additionally, viral infections can induce specific alterations in lipid profiles, as evidenced by observations of diseases caused by viruses, such as Ebola, dengue, Zika, tick-borne encephalitis, hepatitis C, and SARS-CoV-2 (21–26). The rapidly increasing knowledge of the importance of lipids in cell organization, signaling networks, and viral disease outcomes, therefore, led us to investigate the potential perturbations of cellular lipid metabolic networks in human serum to establish and promote SFTSV infection.

To comprehensively delineate the host lipid-virus interaction networks impartially, we conducted a comprehensive lipidomic analysis of human serum samples collected from healthy controls and SFTS patients with mild, severe, and fatal outcomes. Our study revealed global lipidomic alterations that indicated specific metabolic fingerprints following SFTSV infection. Additionally, this study provided evidence that lipids can serve as novel biomarkers for predicting the progression of SFTS, offering new insights into the clinical prognosis and treatment of this disease.

## MATERIALS AND METHODS

### Human subjects and sample collection

Blood specimens were collected from March 2022 to October 2022 from 90 individuals, including 30 healthy controls and 60 SFTS patients who presented at Nanjing Drum Tower Hospital, Nanjing, China, at the time of enrollment in the study. Clinically relevant medical information was collected at the time of enrollment from the subject or from the medical records. All participants recruited in this study provided written informed consent.

The clinical cohort consisted of SFTS participants (33 females and 27 males) with confirmed SFTSV infection by RT-qPCR and healthy subjects (14 females and 16 males). Patients who met the following exclusion criteria were excluded from the study: (i) had other infectious diseases; (ii) had negative RT-qPCR results for SFTSV infection; (iii) had previously been diagnosed with chronic diseases, such as diabetes or cardiovascular disease; (iv) had other lipid dysfunction diseases; and (v) had hematologic disorders, such as leukemia and thrombocytosis. SFTS patients were categorized into mild, severe, and fatal groups based on the diagnostic criteria for clinical outcomes. Specifically, a mild group of SFTS patients displayed mild symptoms with temperature or no reduction in platelets. Severe cases were defined as patients meeting at least one of the following criteria: multiorgan dysfunction, acute respiratory distress syndrome (ARDS), sepsis, disseminated intravascular coagulation (DIC), failure of one or more organs (such as heart failure, acute renal failure, or liver failure), or infection-induced toxic shock. The fatal group consisted of SFTS patients with fatal clinical outcomes. The detailed clinical characteristics of the patients in the research cohort are listed in Table 1. The serum was obtained through centrifugation at 1,500 rpm for 15 min and stored in a −80°C freezer until use.

**TABLE 1:** Demographic and clinical characteristics of the study cohort^*a*^

Parameter	Mild patients (*n* = 30)	Severe patients (*n* = 15)	Fatal patients (*n* = 15)	*P* value (mild/severe)	*P* value (mild/fatal)
Male/female (*n*)	13/17	6/9	8/7		
Age (years)	57.8 ± 10.4	68.9 ± 10.0	70.3 ± 8.12	0.0015**	0.0002***
Interval between collection and onset (days）	7.3 ± 2.0	8.9 ± 2.6	5.5 ± 2.8		
Laboratory parameters					
WBC (×10^9^/L)	3.2 ± 1.4	2.8 ± 1.8	3.5 ± 3.4	0.5832	0.7098
PLT (×10^9^/L)	68.6 ± 24.4	54 ± 25.6	33.7 ± 16.6	0.2619	0.0015**
ALT (U/L)	94.9 ± 83.5	96.2 ± 79.4	395.9 ± 622.5	0.974	0.0656
AST (U/L)	150.7 ± 155.3	192.3 ± 112.9	1,276.3 ± 1,971.4	0.5882	0.0299*
ALP (U/L)	96.0 ± 55.5	72.1 ± 21.8	146.6 ± 91.4	0.3655	0.1043
GGT (U/L)	74.4 ± 79.6	93.7 ± 46.0	1,01.8 ± 58.6	0.6156	0.3996
LDH (U/L)	685.6 ± 411.2	953.6 ± 728.7	3,350.9 ± 4,865.3	0.3042	0.0366*
PCT (ng/mL)	0.15 ± 0.14	0.5 ± 0.7	5.0 ± 11.0	0.0878	0.1349
IL-6 (pg/mL)	31.4 ± 57.9	169.6 ± 270.5	2,114.0 ± 5,276.0	0.1034	0.1808
CRP (g/L)	24.3 ± 54.9	90.6 ± 67.9	9.8 ± 8.1	0.0433*	0.4994
INR	1.0 ± 0.1	1.1 ± 0.1	1.3 ± 0.2	0.0086**	0.0001***
PT-sec (s）	11.2 ± 0.7	12.4 ± 1.0	14.4 ± 2.5	0.0085**	0.0001***
APTT (s)	34.6 ± 4.5	38.2 ± 12.6	54.4 ± 14.0	0.3448	<0.0001****
TT (s)	21.1 ± 1.9	39.9 ± 40.8	54.7 ± 52.6	0.0758	0.0186*
BNP (pg/mL)	226.9 ± 454.6	850.8 ± 605.1	946.5 ± 1,178.7	0.0034**	0.1649
TnT (μg/L)	0.03 ± 0.04	0.18 ± 0.2	0.5 ± 0.8	0.0276*	0.0524
CK (U/L)	728.4 ± 873.5	1,042.6 ± 1,702.3	820.8 ± 1,127.3	0.6165	0.9214
CK-MB (U/L)	22.9 ± 21.1	17.6 ± 9.3	29.6 ± 18.8	0.6383	0.4781

^*a*^ The data are presented as means ± standard deviations (SD). WBC, white blood cell; PLT, platelets; ALT, alanine aminotransferase; AST, asparate aminotransferase; ALP, alkaline Phosphatase; GGT, glutamyltransferase; LDH, lactate dehydrogenase; PCT, procalcitonin; INR, international normalized ratio; PT, prothrombin time; APTT, activated partial thromboplastin time; TT, thrombin time; CRP, C-reactive protein; BNP, brain natriuretic peptide; TnT, troponin T; CK, creatine kinase; CK-MB, creatine kinase MB. Statistics were calculated using the unpaired two-tailed *t*-test for single comparison variables between groups: *P < 0.05; **P < 0.01; ***P < 0.001; ****P < 0.0001.

### Detection and collection of clinical parameters

Blood cell counts, including white blood cell (WBC) and platelet (PLT) counts, were determined by an automated hematology analyzer (Sysmex Corporation, Japan). Clinical and biochemical parameters, such as alanine aminotransferase (ALT), aspartate aminotransferase (AST), lactate dehydrogenase (LDH), C-reactive protein (CRP), platelet (PLT), procalcitonin (PCT), brain natriuretic peptide (BNP), troponin T (TnT), creatine kinase (CK), and creatine kinase-MB (CK-MB) levels, were measured by a biochemical analyzer (Beckman AU5400, Germany). The prothrombin time (PT), activated partial thromboplastin time (APTT), international normalized ratio (INR), and thrombin time (TT) were detected by a Sysmex CS-5100 automated coagulation analyzer. All the data were collected from the hospital laboratory information system and medical records system.

### Lipid extraction

The frozen serum was thawed at 4°C, and 40 µL of thawed serum was transferred to a 2.0-mL centrifuge tube, followed by the addition of 300 µL of methanol to the tube and vortexing for 1 min. Afterward, 1,000 µL of methyl tert-butyl ether was added to the tube and vortexed for 1 h. Then, 250 µL of ultrapure water was added, and the mixture was vortexed for 1 min. After vortexing, the tube was centrifuged at 12,000 rpm at 4°C for 10 min. After centrifugation, 400 µL of the supernatant was transferred to a 1.5-mL centrifuge tube and evaporated by vacuum centrifugation. The dried pallet was reconstituted in 100 µL of solution mixed with 90 µL of isopropanol/acetonitrile (1:1, vol/vol), 5 µL of *n*-nonadecanoic acid solution (25 µg/mL) and 5 µL of phosphatidylcholine (PC) (12:0/13:0) solution (15.16 µM). PC (12:0/13:0) and nonadecylic acid (19:0) were used as internal standards for semiquantitative analysis in positive mode and negative mode, respectively. The constitution was then centrifuged at 12,000 rpm at 4°C for 10 min, and 90 µL of the supernatant was transferred to a fresh tube for LC-MS/MS analysis. The quality control (QC) sample was prepared by mixing an equal aliquot of the supernatants from all the samples.

### Chromatographic conditions

A UPLC I-Class ultra-performance liquid chromatograph equipped with a Waters UPLC BEH C8 was used for separation at a column temperature of 55°C. The injection volume was 4 µL for both negative and positive ion modes. For the positive volume, mobile phase A consisted of 0.1% formic acid, acetonitrile/water (6:4, vol/vol) solution, and 5 mM ammonium formate, and mobile phase B consisted of 0.1% formic acid, acetonitrile/isopropanol (1:9, vol/vol) solution, and 5 mM ammonium formate. The analysis was carried out with gradient elution at a flow rate of 0.26 mL/min. Elution steps were performed as following: 0–2 min, 100% phase A; 2–9 min, 70% phase A, 30% phase B; 9–11 min, 30% phase A, 70% phase B; 11–12 min, 5% phase A, 95% phase B; 12–14.1 min, 100% phase B; 14.1–16 min, 100% phase A. For the negative volume, mobile phase A consisted of 0.04% acetic acid, an acetonitrile/water (1:10, vol/vol) solution, and 1 mM ammonium acetate, and mobile phase B consisted of an acetonitrile/isopropanol (1:1, vol/vol) solution. The analysis was carried out with gradient elution at a flow rate of 0.3 mL/min. The eution procedure was performed by following steps: 0–3 min, 90% phase A, 10% phase B; 3–6 min, 65% phase A, 35% phase B; 6–8 min, 15% phase A, 85% phase B; 8–11.1 min, 100% phase B; 11.1–13 min, 90% phase A, 10% phase B.

### LC-MS/MS analysis and lipid identification

The LC-MS/MS data were collected by a hybrid quadrupole orbitrap mass spectrometer (Q Exactive) equipped with a HESI-II spray probe. The parameters were set as follows: positive ion source voltage, 3.7 kV; heated capillary temperature 320°C; sheath gas pressure 30 psi; auxiliary gas pressure 10 psi; and desolvation temperature 300°C. Both the sheath gas and the auxiliary gas were nitrogen. The collision gas was also nitrogen with a pressure of 1.5 mTorr. The parameters of the full mass scan were set as follows: resolution 7,000, autogain control target 1 × 10^6^; maximum isolation time 50 ms; and *m*/*z* range 150–1,500. The mass axis was calibrated by an external standard method with a mass tolerance of 5 ppm. The calibrated *m*/*z* values were 74.09643, 83.06037, 195.08465, 262.63612, 524.26496, and 1,022.00341 for positive mode and 91.00368, 96.96010, 112.98559, 265.14790, 514.28440, and 1,080.00999 for negative mode, respectively. We used the MS/MS data for molecular identification. Specifically, the human metabolome database and lipid maps were used to match the primary molecular weight. The metabolites were then characterized by the dd-MS2 scan mode with the following parameters: resolution 17,500, auto gain control target 1 × 10^5^, maximum isolation time 50 ms, loop count of top 10 peaks, isolation window of *m*/*z* 2, collision energy 30 V, and intensity threshold 1 × 10^5^. A Xcalibur 2.2 SP1.48 software was used to control the LC-MS/MS system and collect data.

The human metabolome database and lipid maps were used to match the primary molecular weight to identify metabolites. To make sure the accurate identification of the unique lipids from MS/MS spectra, both public database retrieval and manual validation were applied. First, as previously described (27–29), the lipids were identified by matching the parent ion and fragment ions via the Progenesis QI version 3.0 (Waters Corporation) using the public databases including Human Metabolome Database (HMDB) and Lipid Metabolites and Pathways Strategy Database (LIPID MAPS). Subsequently, the lipid identification was further validated by considering the mass error of mother ions (<5 ppm), fragment score (>40), and isotope distribution similarity (>80) as well as retention behavior.

### Data processing

The collected data were processed by Progenesis QI with the steps of raw data introduction, peak alignment, peak extraction, adduct deconvolution, and normalization to obtain a table consisting of retention time, *m*/*z,* and peak intensity. The samples with the most signals in QC were selected as the benchmark, and the remaining samples were peak aligned. For the peak extraction, positive mode retention time was 0.7–16 min, negative mode retention time was 0.7–13 min, and the peak width was greater than 0.05 min. The various adduct ions (such as hydrogen adduct ions and sodium adduct ions) were deconvoluted for each ion feature. In addition, the ion features with a variation coefficient >15% in the quality control samples were eliminated to obtain reliable and reproducible metabolites. The processed data are included in Dataset S1 and Dataset S2.

The orthogonal partial least squares discriminant analysis (OPLS-DA) model was used to distinguish global lipid changes between the SFTS patients and HC samples. The variable importance in the projection (VIP) value of the OPLS-DA model (VIP > 1) and the *P*-value of the *t*-test (*P* < 0.05) was used to identify differentially expressed lipids. For volcano plot analysis, differentially abundant metabolites were analyzed through *P*-values (*P* < 0.05) and absolute log2fold changes (|log2FC| ≥ 1.0).

The identified metabolites were annotated using Kyoto Encyclopedia of Genes and Genomes (KEGG) compound database. And annotated metabolites were then mapped to KEGG pathway database. Pathways with significantly altered metabolites mapped to were applied to metabolite sets enrichment analysis (MSEA), and their significance was examined by hypergeometric test’s *P*-values.

### Statistical analysis

The normal distribution of lipids in each group was analyzed by the Shapiro-Wilk test. Statistical analysis of clinical parameters between two groups was carried out by unpaired two-tailed *t*-tests when normally distributed. One-way analysis of variance (ANOVA) was used to compare more than two groups. A *P*-value less than 0.05 was considered statistical significance (*P* < 0.05). Pearson analysis was employed to analyze the correlation between lipids and clinical parameters, with correlations considered significant if the *r*-value was greater than 0.2 and the *P*-value was less than 0.05 (*r* > 0.2, *P* < 0.05).

Receiver operating characteristic (ROC) curve analysis was performed using MedCalc version 22.009 software (MedCalc Software Ltd., Belgium). Variables with *P*-values < 0.05 in the univariate analysis were used for multivariate logistic regression analysis. The diagnostic ability of the predictive indexes for patient prognosis was evaluated, and the areas under the ROC curves (AUROCs) were calculated.

## RESULTS

### Experimental design and clinical characteristics

**Fig 1:**
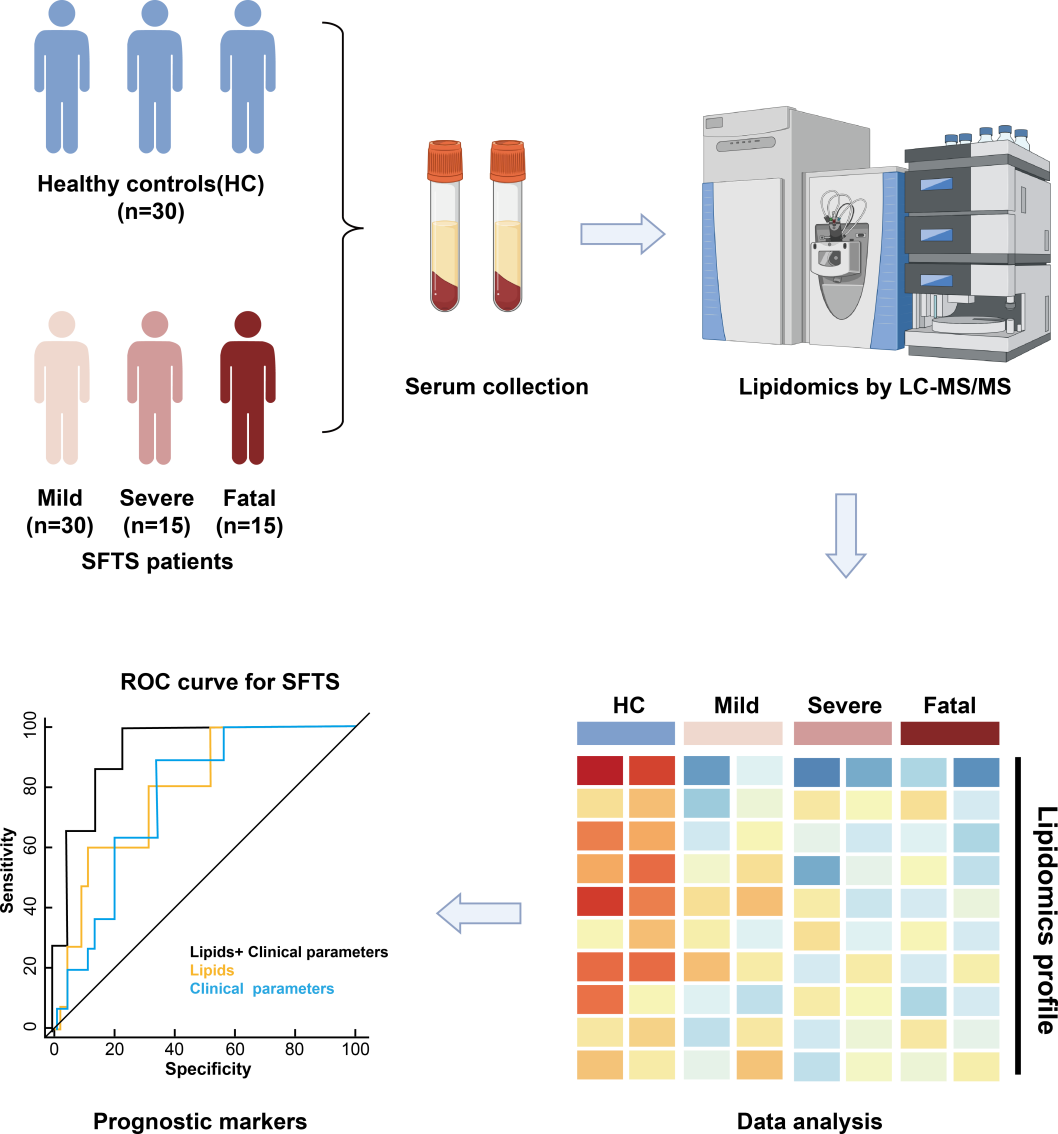
The study design of lipidomic analysis of serum samples from SFTS patients vs healthy controls.

To understand how the host lipid metabolism is altered by SFTSV infection in human serum samples, we conducted global lipidomics using LC-MS/MS to analyze serum samples collected from 60 SFTS patients and 30 healthy donors (Fig. 1). The 60 SFTS patients included 30 mild patients, 15 severe patients, and 15 fatal patients. The detailed demographic and clinical characteristics of the patients are summarized in Table 1. Both fatal and severe SFTS patients were significantly older than mild SFTS patients. Furthermore, profound perturbations in clinical parameters, including inflammatory markers (i.e., WBC and CRP), liver dysfunction (i.e., ALT, AST, and LDH), blood coagulation (i.e., INR, PT, APTT, and TT), and myocardial function (i.e., TnT), were detected in severe and fatal SFTS patients, as compared to mild SFTS patients. Taken together, consistent with our previous report (15) and others (30), thrombocytopenia as well as dysfunction of blood coagulation, liver, and heart dynamically progressed in association with disease aggravation.

### The dramatic alteration in the global lipid landscape in the serum of SFTS patients

**Fig 2:**
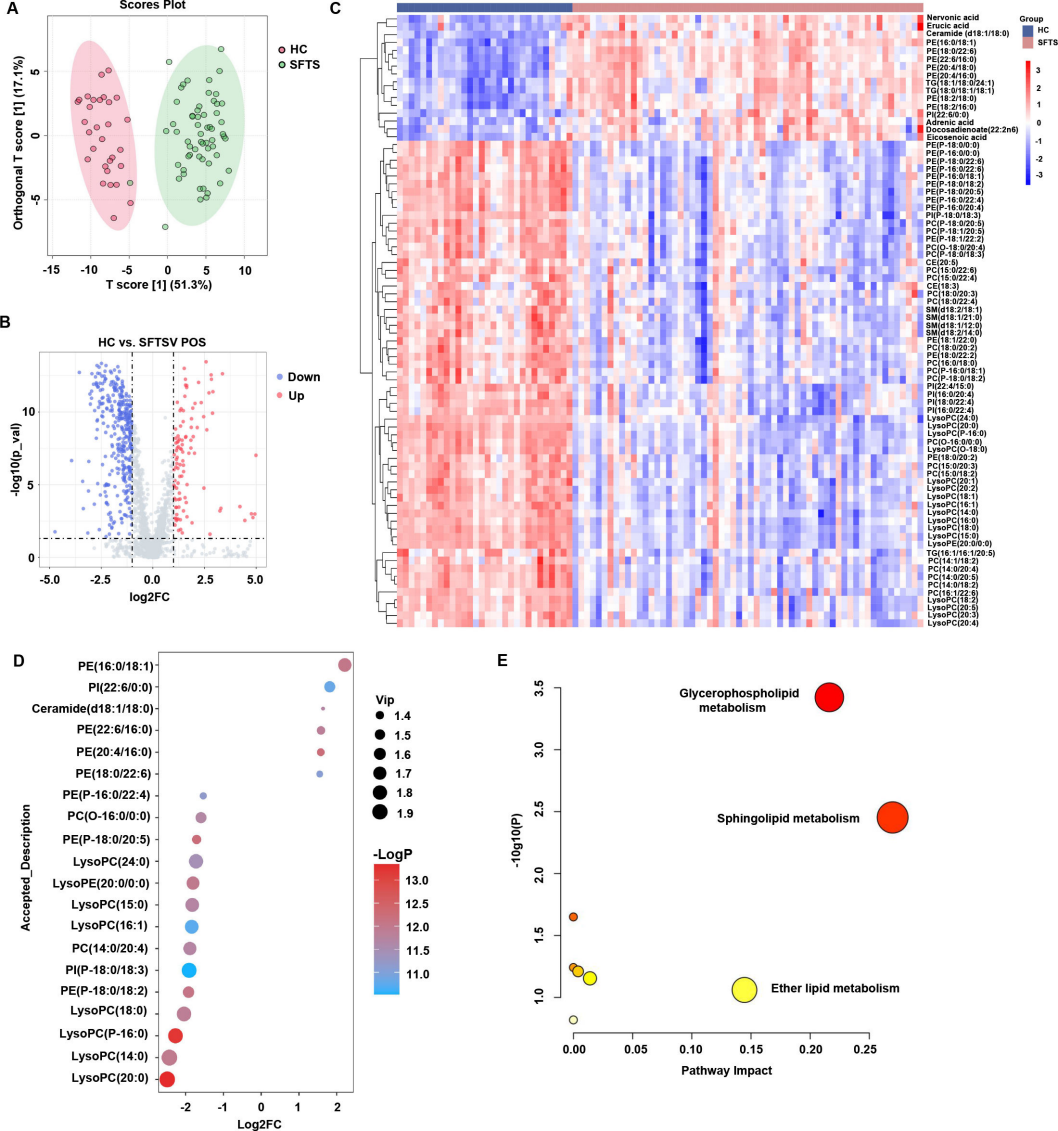
The dramatic alteration of global lipid landscape in serum from SFTS patients. (**A**) Orthogonal partial least-squares discriminant analysis (OPLS-DA) plot showing the pattern of distribution of lipids between SFTS patients and healthy control subjects. VIP > 1 and *P* < 0.05 were used to search for differentially expressed metabolites. (**B**) Volcano plot showing the significantly altered metabolites between SFTS patients and healthy controls (|log2FC| ≥ 1.0). Metabolites that have been significantly changed are highlighted in red for upregulation and blue for downregulation. (**C**) Heatmap shows the changes of metabolites between SFTS patients and healthy control subjects (VIP > 1 and *P* < 0.05). (**D**) The bubble diagram shows the top 20 lipid metabolites between SFTS patients and healthy controls. (**E**) KEGG pathway showing the enrichment of differentially expressed lipids in multiple metabolic pathways between SFTS patients and healthy control subjects. The *x*-axis represents the value calculated from the pathway topology analysis. The *y*-axis represents the transformation of the −log10 (p) calculated from the enrichment analysis, the node color is based on its −log10 (p) value, and the node size reflects the pathway impact values.

To explore the biological significance of lipid metabolites in serum samples from SFTS patients, we investigated the global lipid landscape by applying an untargeted lipidomics approach to human serum from SFTS patients and healthy subjects. We identified a total of 2,363 unique lipid metabolites in positive mode and 1,751 in negative mode. Orthogonal partial least-squares discriminant analysis (OPLS-DA) revealed that the lipids were separated between SFTS patients and healthy controls (Fig. 2A). Notably, there is one mild SFTS patient sample at the convalescent stage that clusters with healthy control samples. Compared with those in healthy donors, lipids whose expression significantly changed (|log2FC| ≥ 1.0) are shown in the volcano plot, in which the expression of 114 metabolites increased and that of 343 metabolites decreased in SFTS patients (Fig. 2B). According to the variable importance in projection (VIP) scores (VIP > 1, *P* < 0.05), the heatmap showed that 16 lipids were significantly increased, and 62 lipids were markedly decreased in SFTS patients, as compared to healthy donors (Fig. 2C). The top 20 changed lipids are shown in the bubble diagram (Fig. 2D). In detail, the subclass lipids of PE, specifically, PE (16:0/18:1), PE (22:6/16:0), PE (20:4/16:0), and PE (18:0/22:6), the lipid class of ceramide, and PI were significantly increased, whereas the subclass lipids of PE, specifically, PE (P-18:0/18:2), PE (P-16:0/22:4), and PE (P-18:0/20:5), the lipid class of PC, LysoPC, and LysoPE were remarkably decreased in SFTS patients. The dynamic changes in the identified differential lipids are summarized in Fig. S1. The KEGG pathway analysis revealed that the altered lipids were enriched mainly in the glycerophospholipid metabolism, sphingolipid metabolism, and ether lipid metabolism pathways (Fig. 2E). Together, our findings revealed a global alteration in lipidomics induced by SFTSV infection, suggesting systemic lipid reprogramming in SFTS patients.

### The lipidomic features between mild and severe SFTS patients

**Fig 3:**
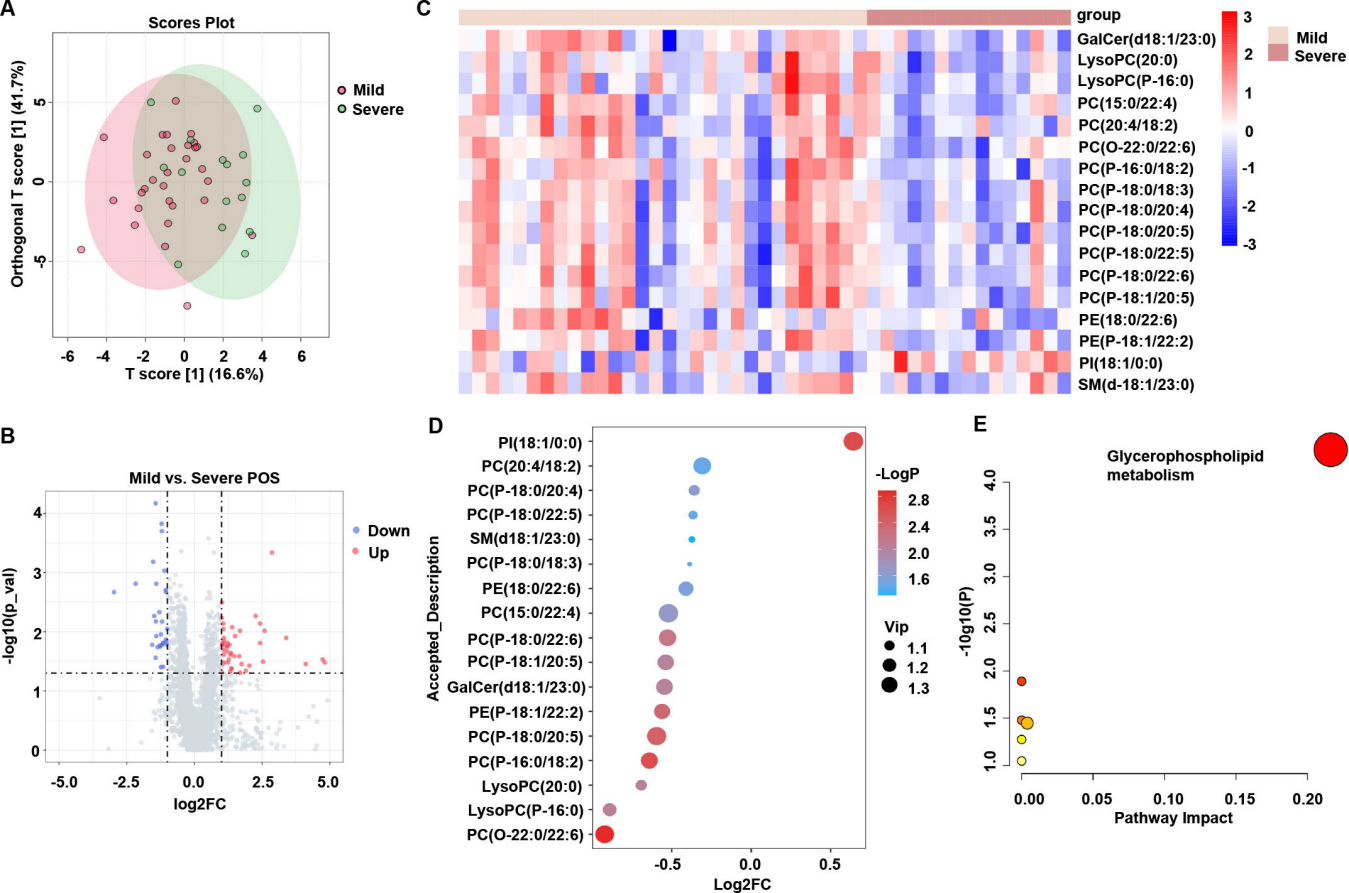
The lipidomic features between mild and severe SFTS patients. (**A**) Orthogonal partial least-squares discriminant analysis (OPLS-DA) plot showing the pattern of distribution of lipids between mild and severe SFTS patients (VIP > 1 and *P* < 0.05 were used to search for differentially expressed metabolites). (**B**) Volcano plot presents the significant change of the metabolites between mild and severe SFTS patients (|Log_2_FC| ≥ 1.0). Metabolites that have been significantly changed are highlighted in red for upregulation and blue for downregulation. (**C**) Heatmap showing the changes of metabolites between mild and severe SFTS patients (VIP > 1 and *P* < 0.05). (**D**) Bubble diagram showing the metabolism of the top 20 lipids between mild and severe SFTS patients. (**E**) KEGG pathway shows the enrichment of differentially expressed lipids in multiple metabolic pathways between mild and severe SFTS patients. The *x*-axis represents the value calculated from the pathway topology analysis. The *y*-axis represents the transformation of the original *P*-value calculated from the enrichment analysis.

To characterize the serum lipid metabolite reprogramming profiles among SFTS patients with disease severity, we first conducted a comparative analysis of lipid profile changes in mild vs severe SFTS patients. Although OPLS-DA showed less separation of lipids between the mild group and severe group in SFTS patients (Fig. 3A), there were 51 increased lipid levels and 30 reduced lipid levels in mild SFTS patients compared to severe donors (Fig. 3B). Furthermore, the heatmap revealed notably perturbed lipids (Fig. 3C), most of which were decreased, including the majority of lipid classes of PC and LysoPC, whereas PI (18:1/0:0) increased (Fig. 3D). The detailed alterations in lipids between mild and severe SFTS patients are summarized in Fig. S2. Furthermore, KEGG pathway analysis revealed that the glycerophospholipid metabolism pathway was the most affected metabolic pathway during the progression from mild to severe symptoms of SFTSV infection, highlighting that glycerophospholipid metabolism might play a key role in the progression of SFTS (Fig. 3E).

### The lipidomic features between mild and fatal SFTS patients

**Fig 4:**
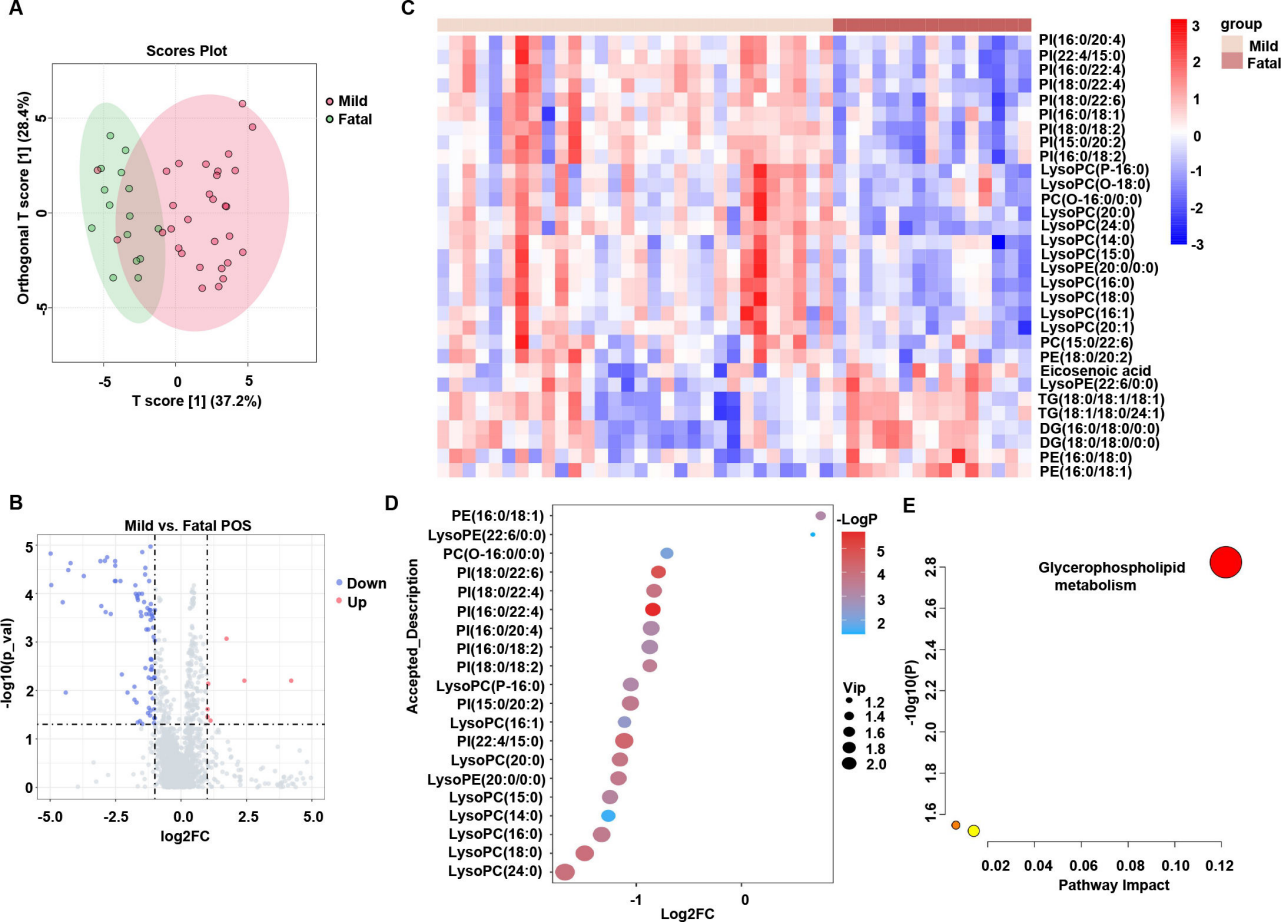
The lipidomic changes between mild and fatal SFTS patients. (**A**) Orthogonal partial least-squares discriminant analysis (OPLS-DA) plot showing the pattern of distribution of lipids between mild and fatal SFTS patients (VIP > 1 and *P* < 0.05 were used to search for differentially expressed metabolites). (**B**) Volcano plot showing the significant changes of the metabolites between mild and fatal SFTS patients (|Log_2_FC| ≥ 1.0). Metabolites that have been significantly changed are highlighted in red for upregulation and blue for downregulation. (**C**) Heatmaps show the different expression patterns of lipids between mild and fatal SFTS patients (VIP > 1 and *P* < 0.05). (**D**) Bubble diagram shows the top 20 lipids metabolism between mild and fatal SFTS patients. (**E**) KEGG pathway showing the enrichment of differentially expressed lipids in multiple metabolic pathways between mild and fatal SFTS patients. The *x*-axis represents the value calculated from the pathway topology analysis. The *y*-axis represents the transformation of the original *P*-value calculated from the enrichment analysis.

We further compared the lipid metabolic expression patterns between mild and fatal SFTS patients. OPLS-DA analysis distinguished severe serum samples from mild serum samples, indicating that the lipidom of fatal SFTS patients underwent significant remodeling (Fig. 4A). Volcano plots revealed that 7 lipids were increased, and 78 lipids were decreased in fatal SFTS patients compared with mild SFTS patients (Fig. 4B). A heatmap was generated to visualize the different expression patterns of lipids (Fig. 4C). Bubble diagram showing the 20 lipids with the greatest changes between mild and fatal SFTS patients. Compared to mild patients, the levels of lipid class LysoPC, LysoPE, PI, PC were significantly decreased, whereas lipid class PE was remarkably increased in fatal patients (Fig. 4D; Fig. S3). KEGG enrichment analysis of these altered lipids revealed that glycerophospholipid metabolism was the most strongly perturbed pathway in patients with fatal disease compared with patients with mild disease (Fig. 4E).

### Disruption of LysoPC (20:0) and LysoPC (P-16:0) in the glycerophospholipid metabolism pathway in SFTS patients with aggravated outcomes

**Fig 5:**
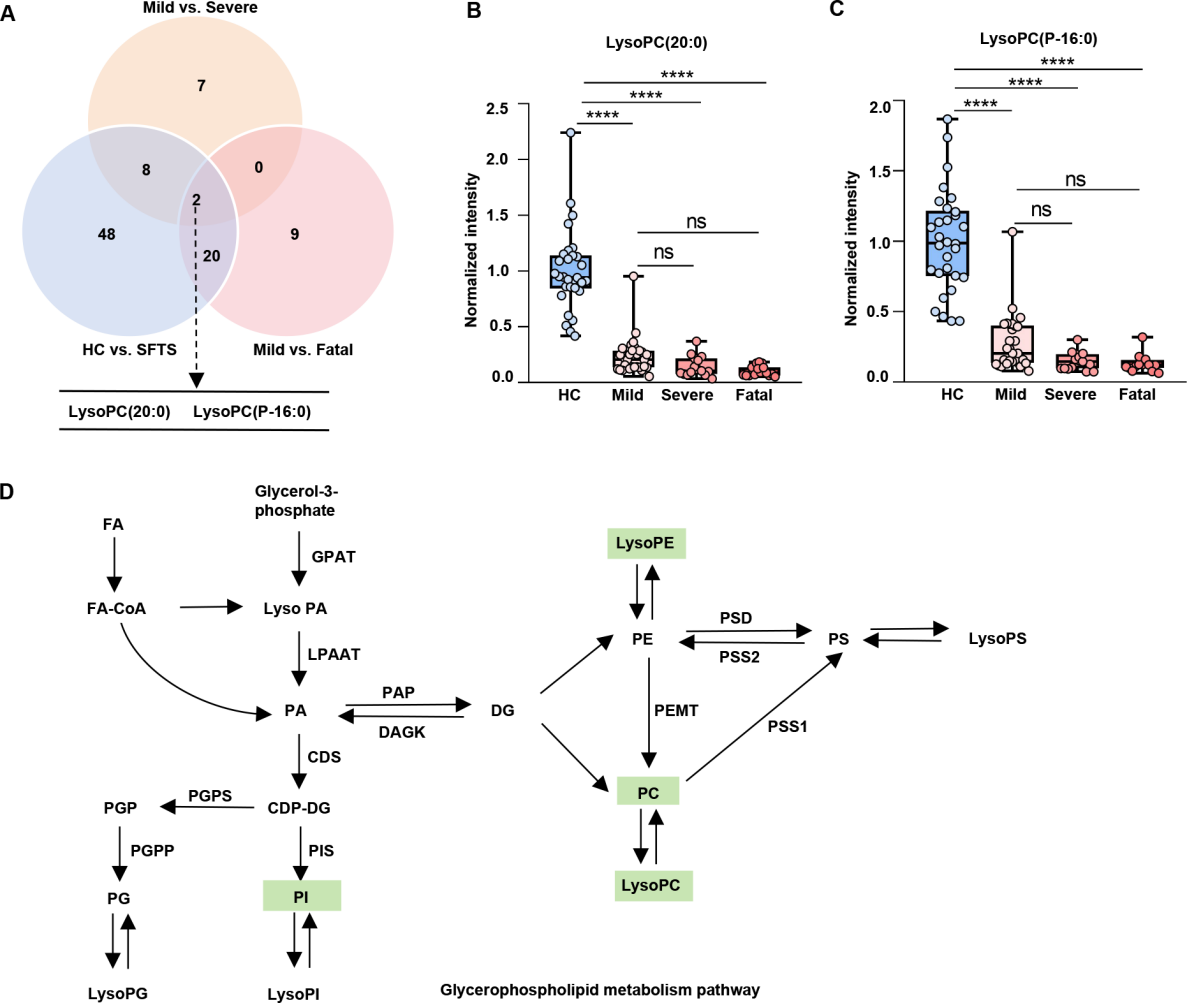
Perturbed LysoPC (20:0) and LysoPC (P-16:0) in glycerophospholipid metabolism pathway among SFTS patients. (**A**) Venn diagram analysis of shared perturbed lipids among mild, severe, and fatal SFTS patients. (**B and C**) Box plots showing the normalized intensities of LysoPC (20:0) and LysoPC (P-16:0) in healthy controls, mild, severe, and fatal SFTS patients. (**D**) Analysis of altered lipids in the glycerophospholipid metabolism pathway constructed from the KEGG pathway database after STFSV infection. Green represents the lipids with decreased expression. Statistical significance was tested by one-way ANOVA. ****, *P* < 0.0001；ns, not significant.

To further investigate the most outstanding metabolism pathway and lipids in SFTSV infection, we conducted a comprehensive analysis. As shown in the Venn diagram, patients with mild disease exhibited differential lipid perturbation compared with patients with fatal and severe disease (Fig. 5A). Notably, we found that two lipids, LysoPC (20:0) and LysoPC (P-16:0), overlapped among the comparison of healthy controls and SFTS patients, mild and fatal SFTS patients, as well as mild and severe SFTS patients. As expected, LysoPC (20:0) and LysoPC (P-16:0) were markedly decreased in SFTS patients compared to healthy controls (4.30-fold, *P* < 0.0001; 4.24-fold, *P* < 0.0001, respectively). Consistently, the levels of these two lipids gradually decreased with the progression of SFTS (Fig. 5B and C). Furthermore, we conducted the ROC analysis of these 20 shared lipids between healthy subjects vs SFTS and mild vs fatal patients identified by the Venn diagram (Fig. S4A). The ROC curves of these 20 lipids revealed good performance, with AUCs greater than 80% in distinguishing HC from SFTS patients (Fig. S4B), and AUCs greater than 70% in distinguishing mild from fatal SFTS patients (Fig. S4C).

Glycerophospholipid metabolism was found to be the most common perturbed pathway in SFTS patients, and LysoPC (20:0) and LysoPC (P-16:0) were also included in this pathway. Glycerophospholipids, as the major constituents of cellular membranes (31), serve as docking platforms for different receptors and play an important role in virus infection (32, 33). To provide an overview of how SFTSV disrupted the glycerophospholipid metabolism pathway, we constructed a lipidomic network map showing the changes in the levels of lipids detected in our study in response to SFTSV infection (Fig. 5D). Specifically, we observed markedly decreased levels of the lipid classes PC, LysoPC, and PI in SFTS patients as indicated by the green shadow in the graph.

### The correlation of LysoPC (20:0) and LysoPC (P-16:0) with the severity of SFTS patients

**Fig 6:**
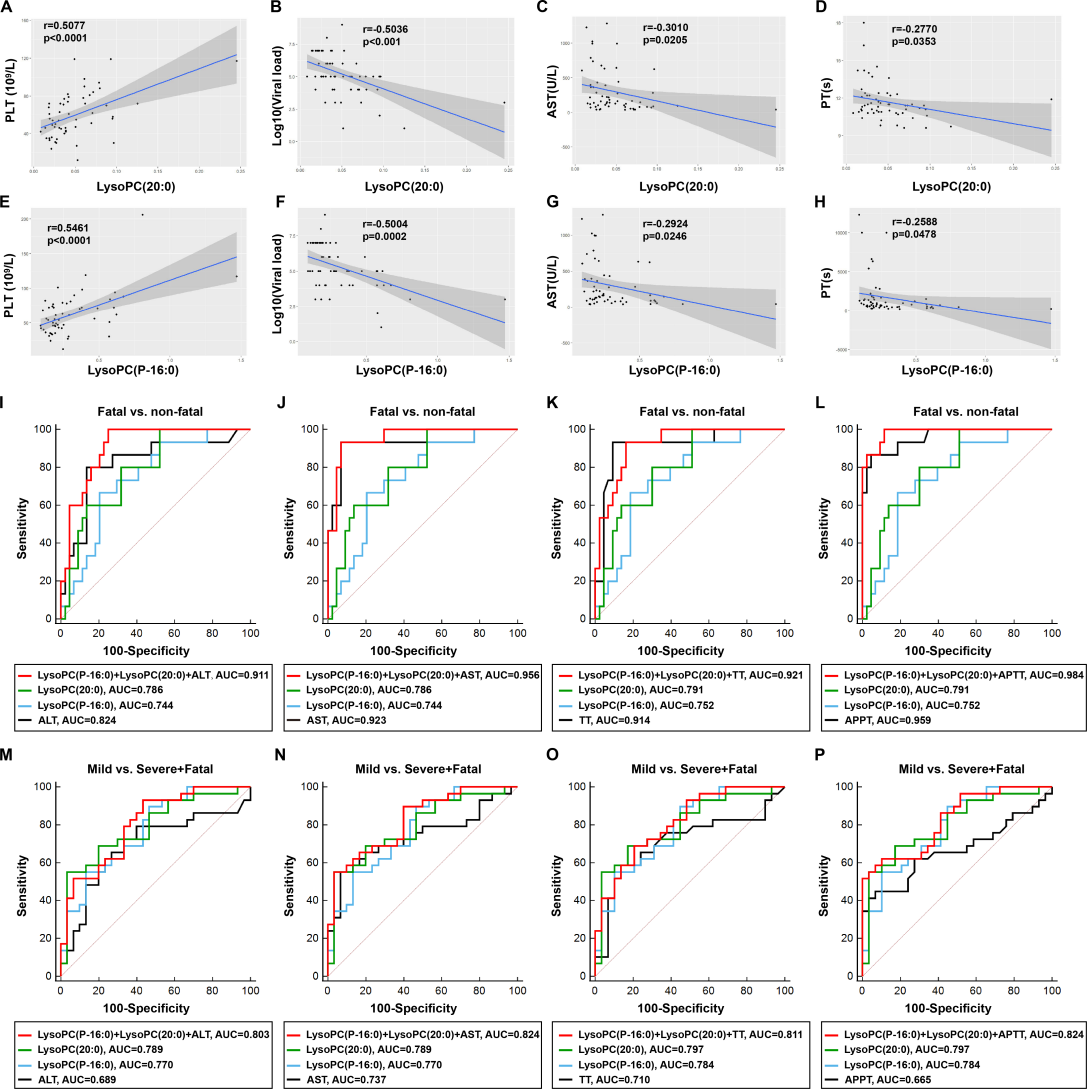
The correlation of LysoPC (20:0) and LysoPC (P-16:0) with the severity of SFTS patients. (**A–D**) Correlation analysis of the serum level of LysoPC (20:0) with the PLT (**A**), viral load (**B**), AST (**C**), and LDH (**D**) in SFTS patients, respectively. (**E–H**) Correlation analysis of the level of serum LysoPC (P-16:0) with the PLT (**E**), viral load (**F**), AST (**G**), and LDH (**H**) in SFTS patients, respectively. (**I–L**) The curve (AUC) of clinical laboratory parameters including ALT (**I**), AST (**J**), TT (**K**), and APTT (**L**) combined with LysoPC (20:0) and LysoPC (P-16:0) to distinguish fatal from nonfatal SFTS patients. (**M–P**) The curve (AUC) of clinical laboratory parameters including ALT (**M**), AST (**N**), TT (**O**), and APTT (**P**) combined with LysoPC (20:0) and LysoPC (P-16:0) to distinguish mild cases from severe and fatal SFTS patients.

To confirm the clinical significance of LysoPC (20:0) and LysoPC (P-16:0) in SFTS patients, a correlation analysis between key perturbed lipids and key clinical laboratory parameters was performed. The level of LysoPC (20:0) was significantly correlated with the PLT (*r* = 0.5077, *P* < 0.0001) and negatively correlated with the viral load (*r* = −0.5036, *P* < 0.001), AST (*r* = −0.3010, *P* = 0.0205), and PT (*r* = −0.2770, *P* = 0.0353), respectively (Fig. 6A through D). Similarly, the level of LysoPC (P-16:0) also was significantly correlated with the PLT (*r* = 0.5461, *P* < 0.0001) and negatively correlated with the viral load (*r* = −0.5004, *P* = 0.0002), AST (*r* = −0.2924, *P* = 0.0246), and LDH (*r* = −0.2588, *P* = 0.0478) (Fig. 6E through H). Overall, there were weak to moderate correlations between these two lipids and key clinical laboratory parameters in SFTS patients. However, there was no significant correlation between LysoPC (20:0) nor LysoPC (P-16:0) and pro-inflammatory factors CRP and IL-6 (Fig. S5). Our data suggested that SFTS-related lipid disturbance is primarily linked to blood coagulation and liver dysfunction, ultimately linking with the disease severity.

To explore the diagnostic performance of LysoPC (20:0) and LysoPC (P-16:0) as biomarkers for SFTS prognosis, we conducted receiver operating characteristic (ROC) curve analysis to assess their ability to distinguish between healthy controls and SFTS patients with different disease progression. Notably, LysoPC (20:0) and LysoPC (P-16:0) exhibited significant diagnostic value with an area under the curve (AUC) values of 99.0% and 98.72%, respectively, between healthy controls and SFTS patients. The combined ROC curve of these two lipids exhibited greater performance with an area under the curve (AUC) of 99.22% between healthy controls and SFTS patients (Fig. S6A). Similarly, we also detected high performance of these two lipids in distinguishing the mild, severe, or fatal patients (Fig. S6B through C).

Moreover, the ROC curve analysis was carried out to evaluate the ability of clinical laboratory parameters in conjunction with LysoPC (20:0) and LysoPC (P-16:0) to predict the clinical outcome of SFTS patients. Strikingly, the combined ROC curves of the clinical laboratory parameters of ALT, AST, TT, and APTT combined with LysoPC (20:0) and LysoPC (P-16:0) revealed superior performance, with AUCs of 91.1%, 95.6%, 92.1%, and 98.4%, respectively, in distinguishing fatal from nonfatal SFTS patients (Fig. 6I through L). Similarly, the ROC curves of LysoPC (20:0) and LysoPC (P-16:0) combined with ALT, AST, TT, and APTT, respecitvely, also showed reasonable performance with AUCs of 80.3%, 82.4%, 81.1%, and 82.4%, respectively, for distinguishing between mild and non-mild SFTS patients (Fig. 6M through P). Together, our results confirmed that LysoPC (20:0) and LysoPC (P-16:0), along with APTT, yielded the highest prognostic accuracy for the fatality of SFTS, outperforming routine clinical parameters.

## DISCUSSION

Viruses infect host cells by interacting with lipid-rich cell membranes, inducing membrane lysis, generating pores, or binding to receptors on cellular membranes. Viruses hijack host lipid metabolism in a variety of venues to support their life cycle. Therefore, understanding the global lipid metabolic reprogramming is urgently needed to elucidate the pathophysiological mechanisms induced by viruses comprehensively. We previously reported that SFTS patients, compared with healthy controls, exhibited significantly lower levels of HDL-c, LDL-c, cholesterol, ApoAl, and ApoB (15). However, the global landscape of lipidomic alterations in SFTS patients with different clinical outcomes still needs to be discovered.

In this study, we systematically investigated the global lipid profile in serum altered by SFTSV infection and attempted to discover novel biomarkers to predict the SFTS severity based on the lipid profiles of human plasma samples collected from mild, severe, and fatal patients using an untargeted lipidomics method. Our results demonstrated a dynamic dysregulation of lipid metabolites in the SFTS patients, with significantly elevated levels of PEs, triglycerides (TGs), ceramides, adrenic acid, docosadieniate, and eicosenoic acid, and dramatic reductions in SM, PI, PC, lysoPC, and CE levels. These results demonstrated that lipid metabolism in human serum was dramatically altered by SFTSV infection.

Indeed, viral infection could confer the dysregulation of lipid metabolism. Specifically, significant elevations of circulating TGs, PEs, and ceramides were reported by West Nile virus infection in both human and mice (34). The plasma lipidome is profoundly altered with increased levels of PS (phosphatidylserine), PE (phosphatidylethanolamine), PG (phosphatidylglycerol), DG (diacylglycerol), and ceramides in fatalities by Ebola virus (25). An increase in sphingolipid levels was observed in both the mouse model and human plasma infected with Zika virus and rotavirus, identifying a critical role of sphingolipid metabolic pathway in virus replication (24, 35). These studies together with our study provide important evidence on alterations in lipid signatures and the mechanisms involved after virus infection.

Here, we revealed that glycerophospholipid metabolism, sphingolipid metabolism, and ether lipid metabolism were severely impaired during SFTSV infection. The subclass of glycerophospholipids, including PC, PE, PI, PG, and PS, constitute major constituents of cell membranes and play various roles in response to viral infections. PCs, mainly synthesized in the liver, are the only subclass of phospholipids which are necessary for the assembly and excretion of lipoprotein. PCs are the most abundant lipid component (up to 70%) of plasma very low density lipoprotein (VLDL) and also make up close to 40% of nascent high-density lipoprotein (HDL) (36, 37). LysoPCs are mainly derived from the turnover of phosphatidylcholine (PC) in the circulation by phospholipase A2 (PLA2). Our study showed remarkably decreased levels of PCs and lysoPCs in human plasma after SFTSV infection, as consistent with the lower HDL levels in SFTS patients reported by our previous study (15). Although it is reported that plasma lysoPCs and PLA2 could serve as inflammation mediators by triggering the inflammation in monocytes and macrophages (38–40), most of these studies used lipid concentrations far away high from the physiological concentration, which is an artificial manipulation condition that could induce inflammation. Nevertheless, most of the more recent studies using mass spectrometry to quantify LysoPCs mainly reported that decreased levels of LysoPCs in plasma are associated with unfavorable disease outcomes. Decreased plasma levels of LysoPCs and PCs have been observed in sepsis (41, 42), cancer (43), dengue (44, 45), and Ebola (25) infection. It is possible that severe liver dysfunction caused by SFTSV, monitored by the clinical parameters, such as AST and ALT, limited its ability to maintain the physiological lipid metabolism, and ultimately inhibited lipid synthesis.

Decreased plasma LysoPC levels were associated with unfavorable disease outcomes, including sepsis mortality (46), in-hospital mortality in pneumonia (47), and chronic kidney disease patients (48). Similarly, our study demonstrated that the altered LysoPC (20:0) or LysoPC (P-16:0) exhibited correlations with key clinical parameters to a certain degree, including liver function, blood coagulation function, and platelet count. ROC analysis revealed superior performance between patients with fatal and non-fatal SFTS, providing supportive evidence that these lipids are promising biomarkers for predicting the progression of SFTSV infection. Therefore, LysoPC (20:0) and LysoPC (P-16:0) could serve as novel prognostic biomarkers that are correlated with the fatality and severity of SFTS clinical outcome.

Beyond the LysoPCs, the elevated levels of TGs and ceramides observed from our study might be also correlated with disease severity. Increased levels of TGs in plasma are probably due to the increased breakdown of adipose tissue induced by virus infections and inflammation, thereby promoting the release of TGs into circulation (49). The increased level of TG is associated with the severity of COVID-19 (50, 51). As a lipid messenger at the heart of sphingolipid metabolism, ceramides play a significant role in the inflammatory response by modulating the production of cytokines and other immune mediators during viral infection. Plasma ceramide concentration was associated with an increased risk of cardiac death outcomes in patients with stable coronary artery disease (52) and respiratory distress symptoms in COVID-19 patients, which might be used for monitoring disease progression (53).

Our study also has several limitations. We identified a global lipid signature of serum after SFTSV infection in humans and aimed to discover potential lipids that could serve as biomarkers for the progression of SFTS. Therefore, a large sample number included in the subgroup of SFTS patients would be preferred to strengthen the diagnostic value of lipids as biomarkers. Longitudinal sample collection is needed to reveal the dynamic changes in the lipid profile associated with the development of SFTSV infection.

### Conclusion

Collectively, our results revealed dynamic changes in the lipidome and further confirmed alterations in the glycerophospholipid metabolism pathway after SFTSV infection in humans. These altered lipids might serve as novel biomarkers for predicting clinical outcomes caused by SFTSV infection. Our findings provide new insights for understanding the mechanisms of SFTS virus infection and indicate that targeting lipid metabolism may serve as a potential therapeutic strategy.

## Data Availability

The data that support the study findings are available upon reasonable request from the corresponding authors.

## SUPPLEMENTAL MATERIAL

Supplemental figures mbio.02628-24-s0001.docx Fig. S1 to S6.

Dataset S1 mbio.02628-24-s0002.csv Data obtained from positive mode.

Dataset S2 mbio.02628-24-s0003.csv Data obtained from negative mode.

ASM does not own the copyrights to Supplemental Material that may be linked to, or accessed through, an article. The authors have granted ASM a non-exclusive, world-wide license to publish the Supplemental Material files. Please contact the corresponding author directly for reuse.
