# Supplementary material for: A global lipid map of severe fever with thrombocytopenia syndrome virus infection reveals glycerophospholipids as novel prognosis biomarkers
Source: mBio. 2024 Nov 13;15(12):e02628-24. doi: 10.1128/mbio.02628-24 (PMC11633121; doi:10.1128/mbio.02628-24)
Supplement: Supplemental figures — Fig. S1 to S6. [file mbio.02628-24-s0001.docx]

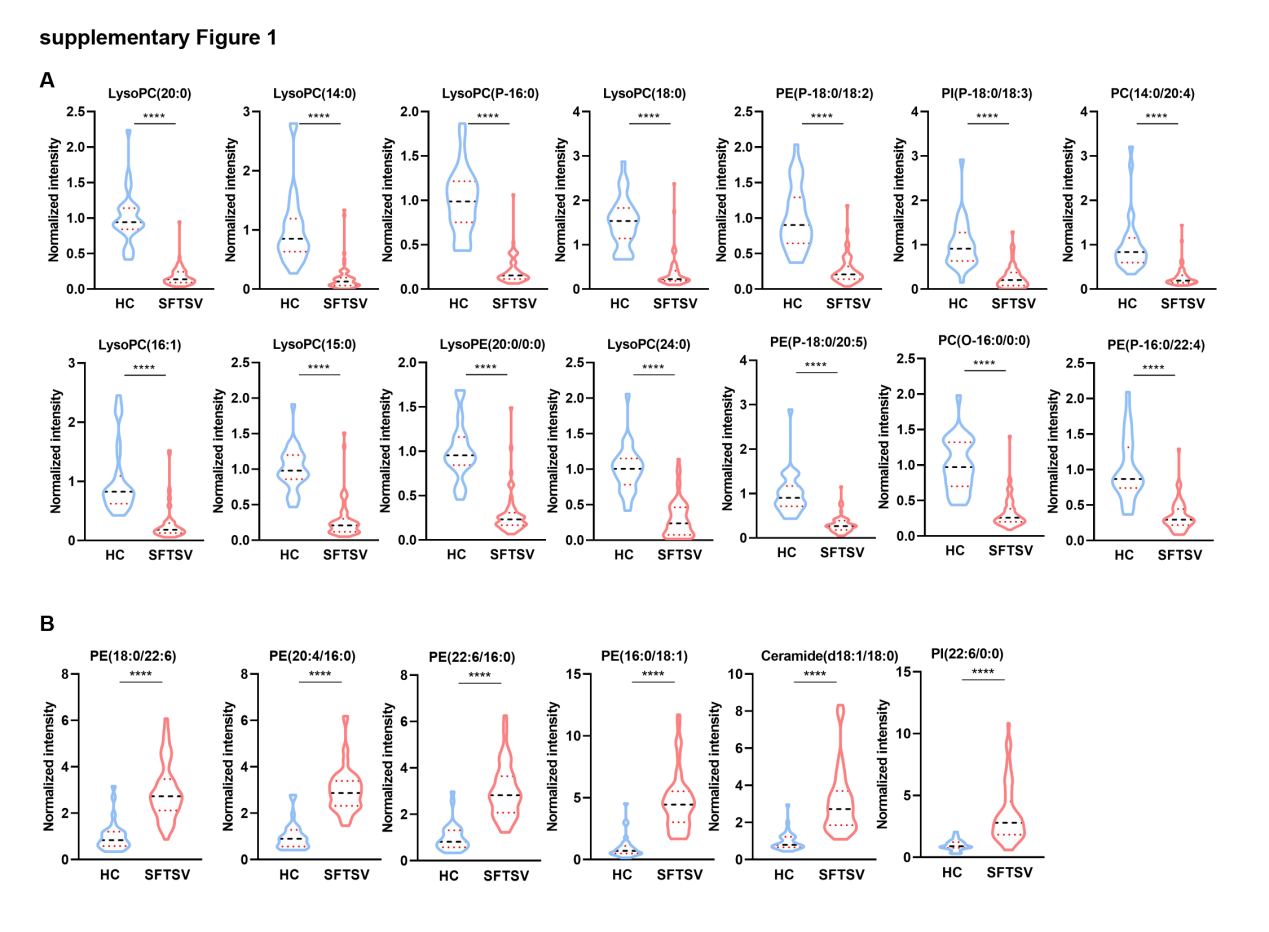


**Figure S1.** **Analysis of the top 20 perturbed lipids between SFTS patient and healthy controls.** **(A)** Violin plot shows the down-regulated lipids in SFTS patient compared with healty controls. **(B)** Violin plot shows the up-regulated lipids in SFTS patient compared with healty controls. The horizontal red and black lines indicate the quartiles and median value of each group, respectively. Statistical significance was tested by two-tailed Student’s t-test . ****, *P* < 0.0001.


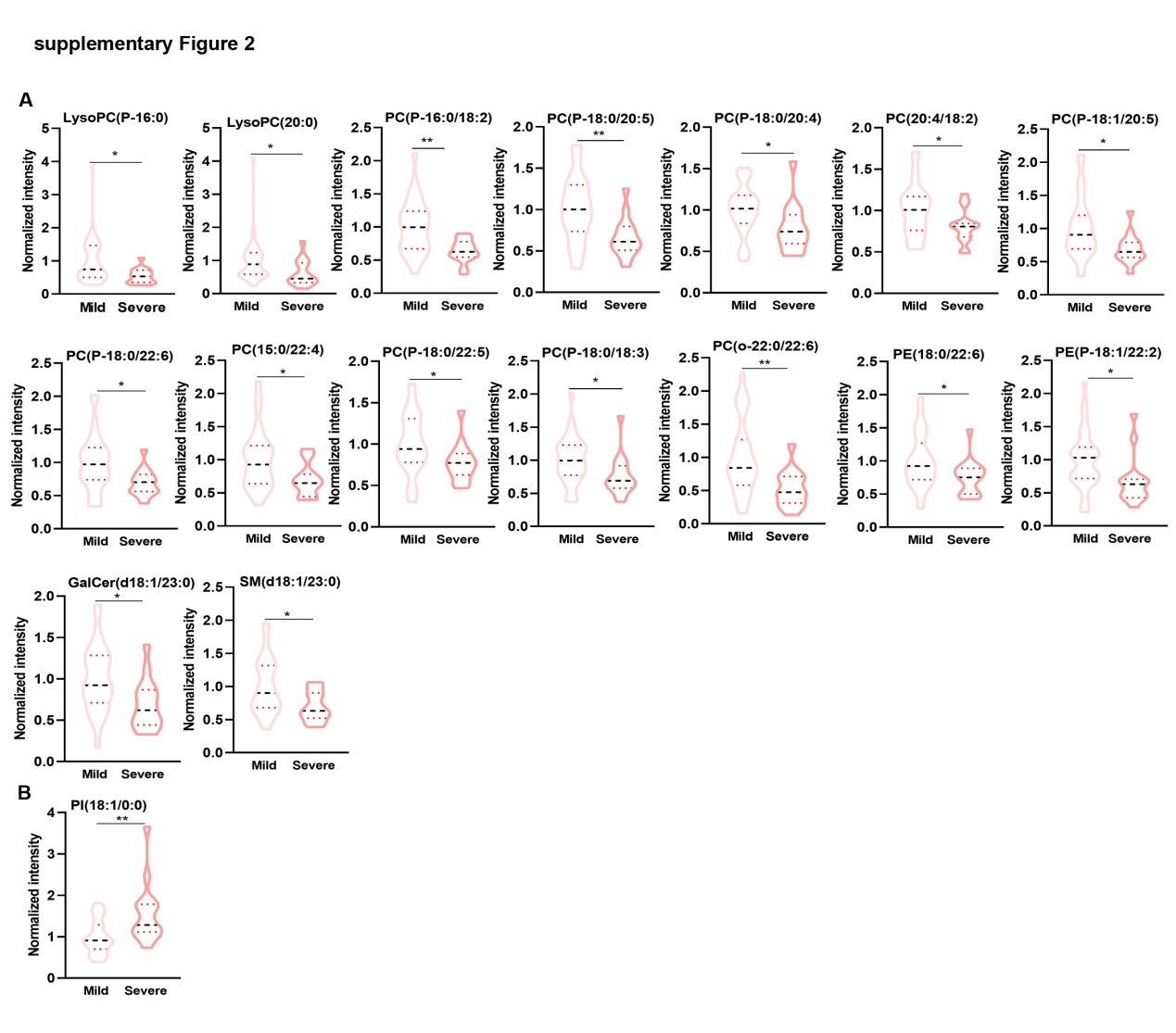


**Figure S2. Analysis of the perturbed lipids between mild and severe SFTS patients. (A)** Violin plot shows the down-regulated lipids in mild SFTS patient compared to severe SFTS patients. **(B)** Violin plot shows the up-regulated lipids in mild SFTS patient compared to severe SFTS patients. The horizontal red and black lines indicate the quartiles and median value of each group, respectively. Statistical significance was tested by two-tailed Student’s t-test. *, *P* < 0.05; **, *P* < 0.01.


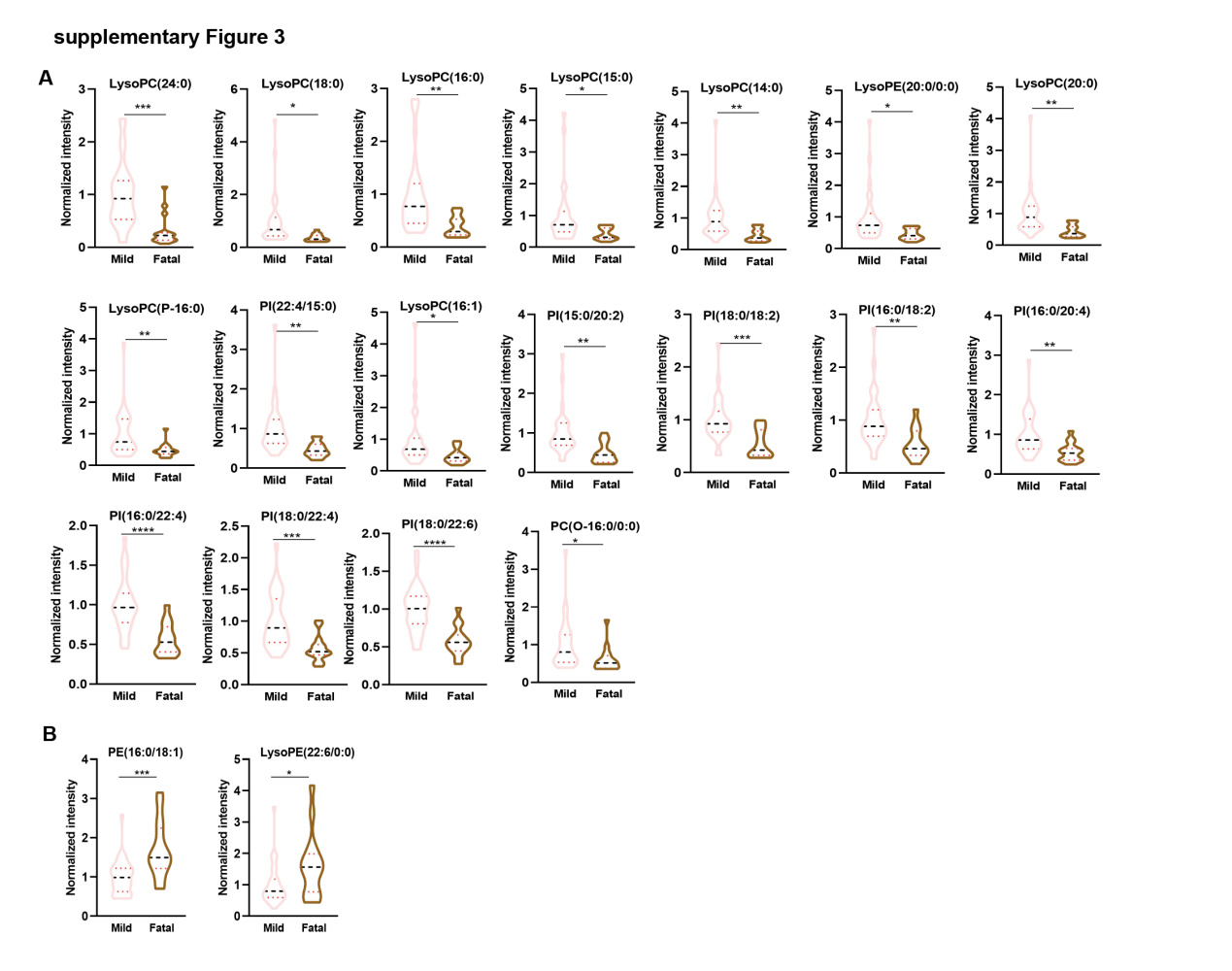


**Figure S3. Analysis of the perturbed lipids between mild and fatal SFTS patients.** **(A)** Violin plot shows the down-regulated lipids in mild SFTS patient compared to fatal SFTS patients. **(B)** Violin plot shows the up-regulated lipids in mild SFTS patient compared to fatal SFTS patients. The horizontal red and black lines indicate the quartiles and median value of each group, respectively. Statistical significance was tested by two-tailed Student’s t-test. *, *P* < 0.05; **, *P* < 0.01; ***, *P* < 0.001.


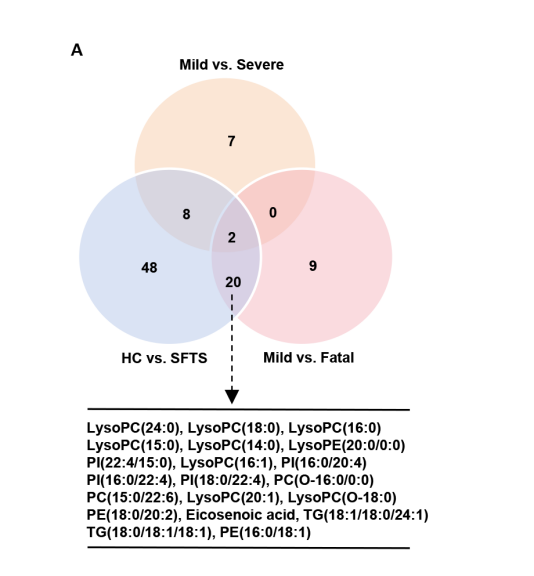

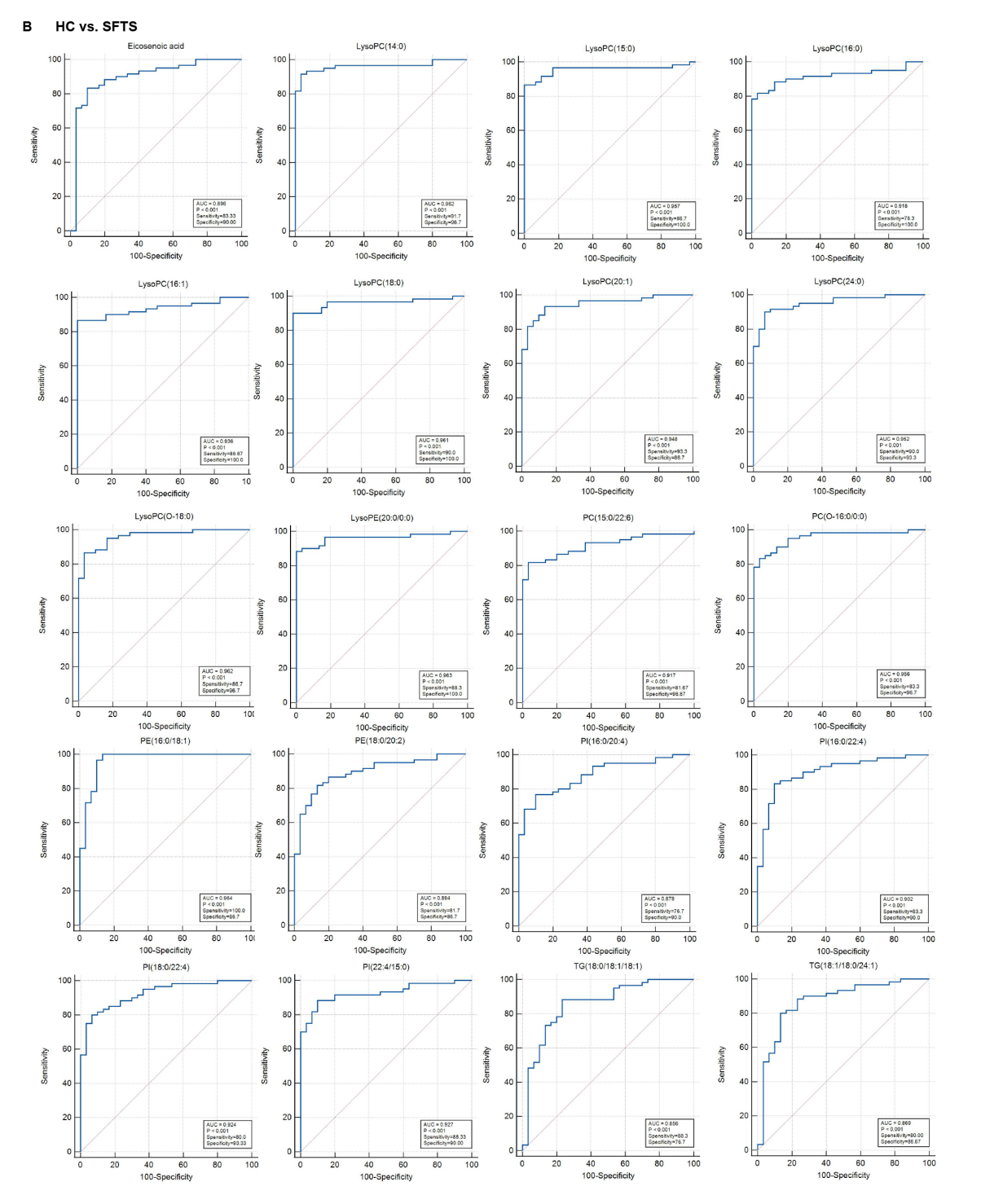

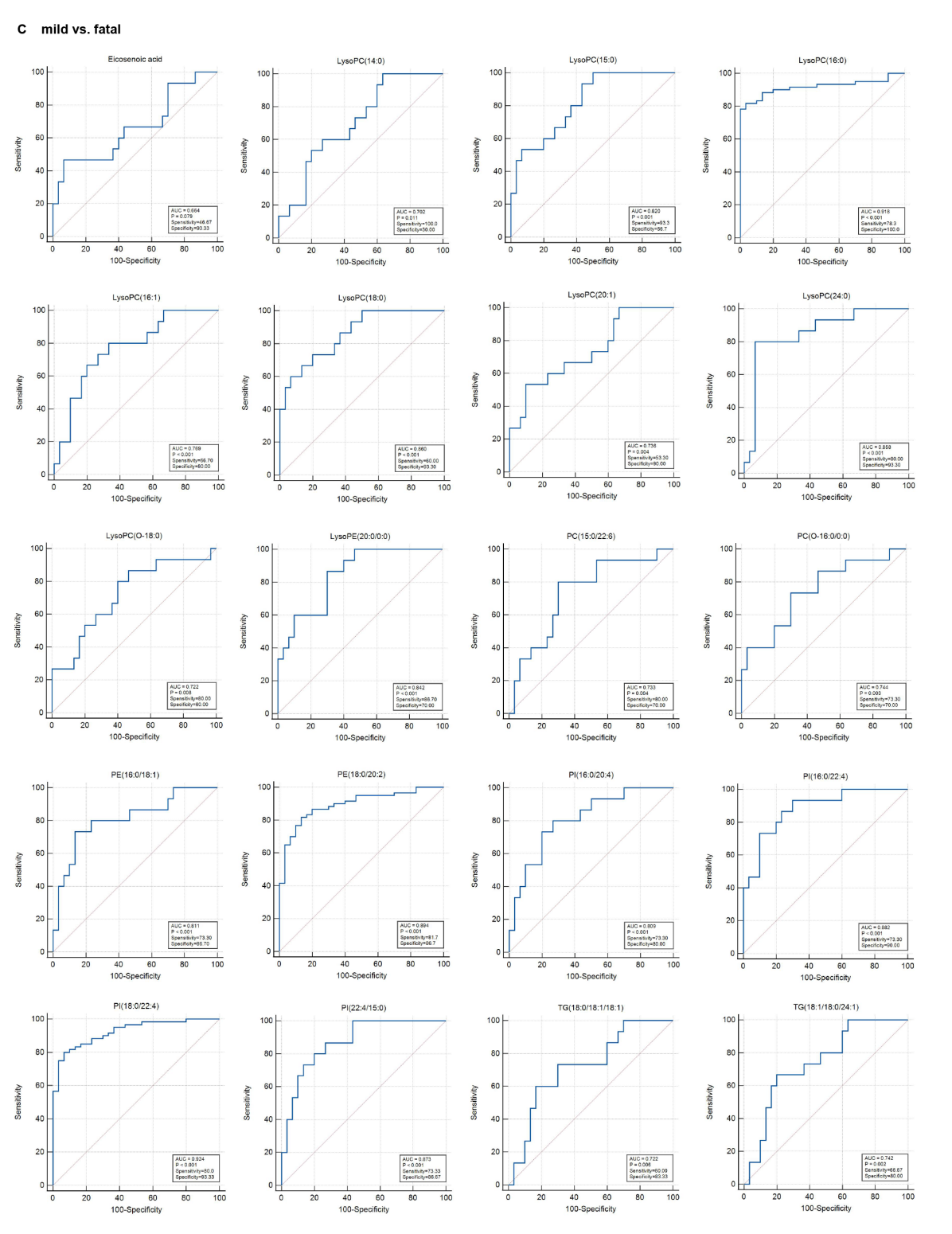


**Figure S4. The shared 20 lipids** **between healthy subjects and SFTS patients as well as between mild and fatal SFTS patients.** **(A)** Venn diagram analysis shows shared perturbed lipids between HC vs. SFTS patients and Mild vs. Fatal patients. **(B)** The curve (AUC) of lipids in distinguishing between healthy subjects (HC) and SFTS patients. **(C)** The curve (AUC) of lipids in distinguishing mild and fatal SFTS patients.


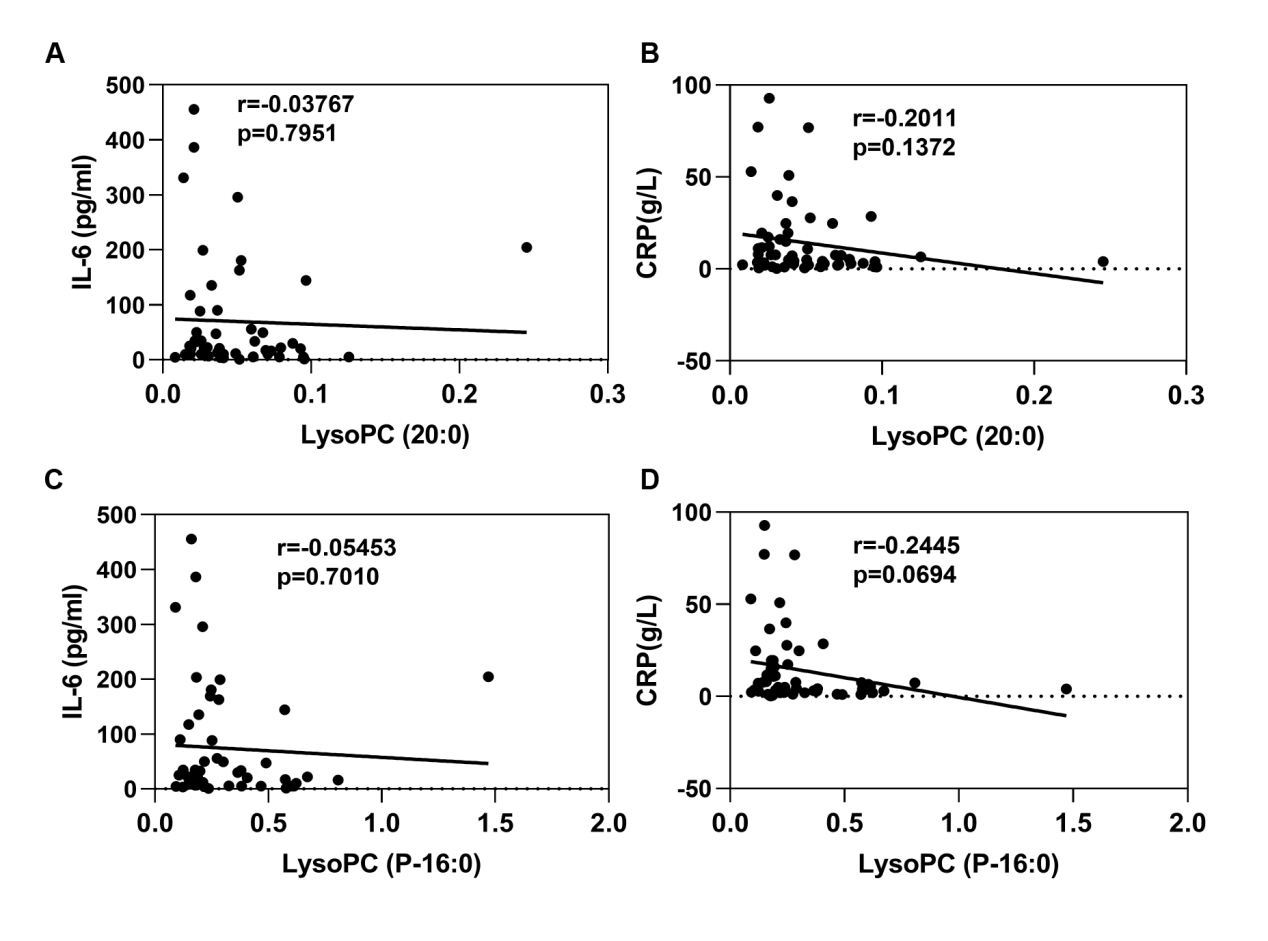


**Figure S5. There was no significant correlation between LysoPC (20:0) nor LysoPC (P-16:0) with the IL-6 and C-reactive protein (CRP).** **(A-B)** Correlation analysis for the level of serum LysoPC (20:0) with IL-6 (A) and CRP (B), respectively. **(C-D)** Correlation analysis for the level of serum LysoPC (P-16:0) with IL-6 (C) and CRP (D), respectively.


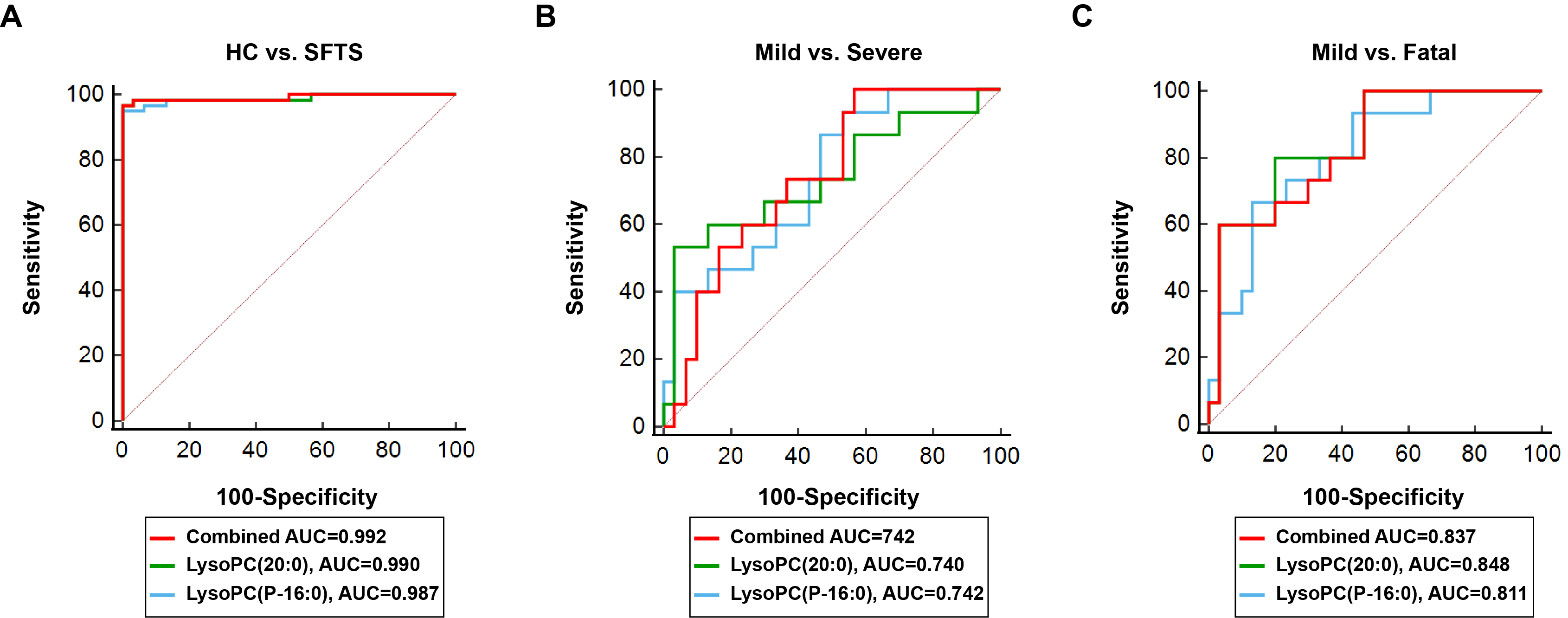


**Figure S6. Analysis of ROC curves of** **LysoPC (20:0) and LysoPC (P-16:0) to distinguish the clinical outcome of SFTS patients.** **(A)** The curve (AUC) of LysoPC (20:0) and LysoPC (P-16:0) in distinguishing healthy donors and SFTS patients. **(B)** The curve (AUC) of LysoPC (20:0) and LysoPC (P-16:0) in distinguishing mild and severe SFTS patients. **(C)** The curve (AUC) of LysoPC (20:0) and LysoPC (P-16:0) in distinguishing mild and fatal SFTS patients.
